# Facilitated Adsorption of Mercury(II) and Chromium(VI) Ions over Functionalized Carbon Nanotubes

**DOI:** 10.3390/toxics11060545

**Published:** 2023-06-20

**Authors:** Gururaj M. Neelgund, Erica A. Jimenez, Ram L. Ray, Mahaveer D. Kurkuri

**Affiliations:** 1 Department of Chemistry, Prairie View A&M University, Prairie View, TX 77446, USA; 2 College of Agriculture and Human Sciences, Prairie View A&M University, Prairie View, TX 77446, USA; 3 Centre for Research in Functional Materials (CRFM), JAIN (Deemed-to-be University), Jain Global Campus, Bengaluru 562 112, Karnataka, India

**Keywords:** carbon nanotubes, polylactic acid, palladium, mercury, chromium, adsorption

## Abstract

By considering the importance of water and its purity, herein, a powerful adsorbent has been developed for the adsorption of two toxic contaminants that commonly exist in water, viz., divalent mercury and hexavalent chromium. The efficient adsorbent, CNTs–PLA–Pd, was prepared by covalent grafting polylactic acid to carbon nanotubes and subsequent deposition of palladium nanoparticles. The CNTs–PLA–Pd could adsorb Hg(II), and Cr(VI) entirely exists in water. The adsorption rate for Hg(II) and Cr(VI) was rapid at initial stage, followed by gradual decrease, and attained the equilibrium. The Hg(II) and Cr(VI) adsorption was perceived within 50 min and 80 min, respectively with CNTs–PLA–Pd,. Further, experimental data for Hg(II) and Cr(VI) adsorption was analyzed, and kinetic parameters were estimated using pseudo–first and second–order models. The adsorption process of Hg(II) and Cr(VI) followed the pseudo–second–order kinetics, and the rate–limiting step in the adsorption was chemisorption. The Weber−Morris intraparticle pore diffusion model revealed that the Hg(II) and Cr(VI) adsorption over CNTs–PLA–Pd occurs through multiple phases. The experimental equilibrium parameters for the Hg(II) and Cr(VI) adsorption were estimated by Langmuir, Freundlich, and Temkin isotherms models. All three models were well suited and demonstrated that Hg(II) and Cr(VI) adsorption over CNTs–PLA–Pd transpires through monolayer molecular covering and chemisorption.

## 1. Introduction

Water is a fundamental need of humans for survival and mandatory for other processes such as domestic, agriculture, and industry. Therefore, pure water is vital for a healthy life. However, excessive industrialization and urbanization are causing water pollution by releasing heavy metals containing effluents. Heavy metals are toxic, bio–accumulative, and naturally non–degradable. Heavy metals’ ingestion at trace levels can also lead to irreparable detrimental impacts such as neurological system disorder, cancer, gastrointestinal disorder, muscular weakness, brain damage, shortness of breath, asthma, hypotension, liver and thyroid damage, allergies, and bone defects [1,2]. The accumulation of heavy metals in the body can cause carcinogenic, teratogenic, and mutagenic effects [3]. Therefore, exposure to heavy metals and their content effluents is not safe. Among different heavy metals, chromium, and mercury are top–priority hazardous because of their acute and chronic detrimental outcomes, which are substantially harmful [1,2]. Chromium is recognized as teratogenic, mutagenic, and carcinogenic [4,5]. It is likely to produce genotoxic DNA effects in the nucleus of cells and can cause cancer, allergies, gastritis, and organ damage [6,7]. Generally, chromium exists with two stable oxidation states, tri and hexavalent, in water [8]. These species differ in physicochemical properties and chemical and biochemical reactivity [8]. Out of both species, trivalent Cr(III) is a biologically essential nutrient that plays a significant role in the metabolism process of glucose, protein, and lipids by enhancing insulin activity [9]. However, hexavalent Cr(VI) is highly poisonous to humans and other living organisms [10]. Generally, Cr(VI) is introduced to the environment by effluents produced by industries like leather tanning, electroplating, metal finishing, textile finishing, steel fabrication, wood preservation, pulp processing, paint, dyes, paper, fertilizers, and photography [11]. Likewise, releasing Cr(VI) content effluents is perilous.

Another heavy metal that has a higher toxicity than chromium is mercury, which is highly volatile and environmentally persistent [12]. In water, mercury exists in the divalent oxidation state as Hg(II) and is significantly hazardous, non–degradable, and bio–accumulative [13]. Hg(II) tends to accumulate in the body tissues, muscles, lipids, liver, and other organs [14]. The accumulation of Hg(II) at the minute level in the body would induce substantial damage to the central nervous, immune, and reproductive systems [13]. It can also cause severe impairment to kidney, renal, lung, and liver function [15]. Hg(II) ions have a high affinity to thiol moieties of enzymes and proteins and could break normal human cells to cause fatal consequences that include organ dysfunction, chronic diseases, metabolic disruption, breakdown of the chromosome, immune and nervous system damage, and may cause death [14,16,17,18,19,20]. Moreover, Hg(II) ions have a high tendency to react with organic and inorganic matter to generate a variety of harmful compounds [21]. The reaction of mercury with organic matter can form the lethal methyl mercury (CH_3_Hg). The CH_3_Hg is exceptionally venomous, has a high binding affinity to proteins, and can accumulate in body tissues to lead to identical consequences like Hg(II) [21]. Hg(II) can enter the environment through myriad anthropogenic sources such as volcanic explosions, weathering of rock, erosion of natural deposits, geothermal processes, wild forest fires, and vaporization from soil and water [14,22]. In addition, it can be introduced by industrial operations like gold refineries, petroleum refineries, electroplating, leather tanning, metal finishing, pharmaceutical industries, and runoff from landfills and croplands [14,23]. Nevertheless, releasing Hg(II) is not innocuous.

Several functional materials have been developed to remove heavy metals from water to prevent their consequences and promote water purification [14,21,24,25,26,27,28,29,30,31]. These materials promoted the removal of heavy metals from water by various techniques like adsorption [32,33], ion exchange [34,35], membrane separation [36], coagulation [37], chemical precipitation [38], extraction [39], dialysis [40], electrochemical separation [41] and more. Among these, adsorption is the most effective, versatile, economically feasible, environmentally sustainable, and technologically promising [1,2,3,7,8,9,10]. The additional advantage of adsorption is its high selectivity and enrichment factor [42]. Because of its importance, several adsorbents such as activated carbon [1], biochar [1], hydroxyapatite/Fe_3_O_4_/polydopamine [2], polyacrylonitrile–based porous carbon [3], vesicular basalt rock [6], activated carbon [7], ferrihydrite [8], exopolysaccharides [9], chitosan/bentonite composite [10], amine–incorporated UIO–66–NH_2_ [20], silica nanoparticles [24], graphene oxide−MnFe_2_O_4_ Magnetic Nanohybrids [25], zinc oxide [26], ZnS [31], and carbon nanotubes [43,44,45] were developed and verified their efficiency in removal of several heavy metals. Compared to these, carbon nanotubes (CNTs) are fascinating due to their unique physical, chemical, and mechanical properties [46,47]. CNTs possess the interesting nanometric one–dimensional structure, high surface–to–volume ratio, high accessible surface area, high aspect ratio, and higher physical and chemical stability [46,47]. These unique properties made CNTs a prospective candidate for adsorption. In addition, the distinct open tubular architecture of CNTs having quantized π electron clouds facilitates the adsorption process [48,49]. Beyond their excellent properties, the application of CNTs in adsorption is hindered due to their hydrophobic nature and the tendency to entangle. To come up with these limitations, functionalization is a suitable approach to transform the hydrophobic CNTs into hydrophilic ones and prevent them from entanglement. It could be attained by grafting the CNTs with a suitable polymer like polylactic acid (PLA). PLA is a vital biopolymer that has crucial properties like non–toxicity, biocompatibility, biodegradability, thermoplasticity, and exceptional mechanical properties required for adsorption [50]. PLA is produced by condensation polymerization of lactic acid, which is derived by the fermentation of sugars from carbohydrate sources such as corn, sugarcane, and tapioca [51]. By considering the importance of CNTs and PLA, herein, CNTs have been functionalized by the covalent grafting of PLA chains. Further, the adsorption capability of PLA–grafted CNTs was improved through the deposition of palladium nanoparticles. Palladium is classically known for its catalytic properties; instead, it has been explored as a potential adsorbent. The efficiency of produced CNTs–PLA–Pd was estimated by the adsorption rate of two toxic pollutants, Hg(II) and Cr(VI), which commonly persist in the water. The adsorption capability, its dynamics, and control mechanisms for the Hg(II) and Cr(VI) adsorption over CNTs–PLA–Pd were evaluated using the pseudo–first and second–order kinetic models. The Weber–Morris intraparticle pore diffusion model was used to find the reaction pathways and the rate–controlling step of the adsorption process. Further, the adsorption equilibrium was determined by fitting the experimental results with Langmuir, Freundlich, and Temkin isotherm models. 

## 2. Experimental Analysis

### 2.1. Materials

The CNTs and chemicals used were purchased from Millipore Sigma. Ultrapure water obtained by the Milli–Q Plus system (Millipore) was used to prepare aqueous solutions.

### 2.2. Preparation of CNTs–PLA–Pd

The CNTs–PLA (10 mg) was prepared according to the reported procedure [52] and dispersed in 7 mL DI water by sonication for 10 min. Then an aqueous solution of PdCl_2_ (5 mL, 0.01 mol L^−1^) was added, and the mixture was stirred at room temperature for 2 h. A freshly prepared 5 mL aqueous solution of NaBH_4_ (0.05 mol L^−1^) was slowly added and stirred at ambient conditions for 4 h. The resulting CNTs–PLA–Pd was centrifuged and purified by washing with DI water. It was dried in a vacuum for 12 h at 40 °C.

### 2.3. Adsorption Experiments 

The stock solution of Hg(II) and Cr(VI) with 1 g L^−1^ was prepared in DI water using mercury(II) chloride (HgCl_2_) and potassium dichromate (K_2_Cr_2_O_7_), respectively. Further, the stock solution was diluted to desired concentrations using DI water. The kinetic adsorption experiments of Hg(II) and Cr(VI) were carried out to find the contact time needed to reach equilibrium. In the typical experiment, 100 mg of CNTs–PLA–Pd was dispersed into 500 mL of Hg(II) and Cr(VI) solution with a concentration of 0.5 mg L^−1^ having an initial pH of 5.7 and 5.1, respectively. It was allowed to stir at room temperature, and an adequate quantity of samples was collected after the required contact time. The adsorbent, CNTs–PLA–Pd was separated by centrifugation, and the concentration of residual Hg(II) and Cr(VI) in the solution was estimated using the atomic absorption spectrometer. The efficiency in Hg(II) and Cr(VI) adsorption as a function of time was monitored for 120 min, and the adsorbed amount of Hg(II) and Cr(VI) by CNTs–PLA–Pd was calculated using the following equation.

(1) $$q_{t}=\frac{(C_{0}-C_{t})\;V}{M}$$

where q_t_ is the amount of pollutant adsorbed (mg/g) at time t; C_0_ is the initial concentration of pollutant in solution (mg L^−1^), and C_t_ is the concentration of pollutant in solution (mg L^−1^) at time t; V is the volume of the solution (L), and M is the amount of adsorbent (g).

Further, the efficiency of CNTs–PLA–Pd towards Hg(II) and Cr(VI) adsorption was determined by:

(2) $$\mathrm{Removal}\;\mathrm{efficiency}\;\left(\%\right)=\frac{(C_{0}-C_{t})\;V}{C_{0}}\times 100$$

For adsorption isotherms experiments, 10 mg of CNTs–PLA–Pd was added to 50 mL Hg(II) and Cr(VI) solution and allowed to stir at room temperature for 24 h to attain equilibrium. The concentration of Hg(II) and Cr(VI) solution was varied from 2 to 10 mg L^−1^ to obtain the adsorption isotherms. After reaching the equilibrium, CNTs–PLA–Pd was separated by centrifugation, and the concentration of Hg(II) and Cr(VI) in the solution was estimated by the atomic absorption spectrometer. The Hg(II) and Cr(VI) adsorption rate at equilibrium, q_e_ (mg g^−1^), was evaluated by:

(3) $$q_{e}=\frac{(C_{0}-C_{e})\;V}{M}$$

where q_e_ is the amount of Hg(II) and Cr(VI) adsorbed (mg/g) at equilibrium.

### 2.4. Characterization

The XRD of samples was obtained using the Scintag X–ray diffractometer, model PAD X, equipped with a Cu Kα photon source (45 kV, 40 mA) at a scanning rate of 3°/min. The ATR–FTIR spectra were perceived by Smith’s ChemID diamond attenuated total reflection (DATR) spectrometer. The SEM images of samples were acquired with the JEOL JXA–8900 microscope, and the X-ray photoelectron spectra (XPS) were acquired by the Perkin Elmer PHI 5600 ci X-ray photoelectron spectrometer. The Hg(II) and Cr(VI) concentration was measured using a Varian SpectrAA 220FS atomic absorption spectrometer.

## 3. Results and Discussion

**Figure 1:**
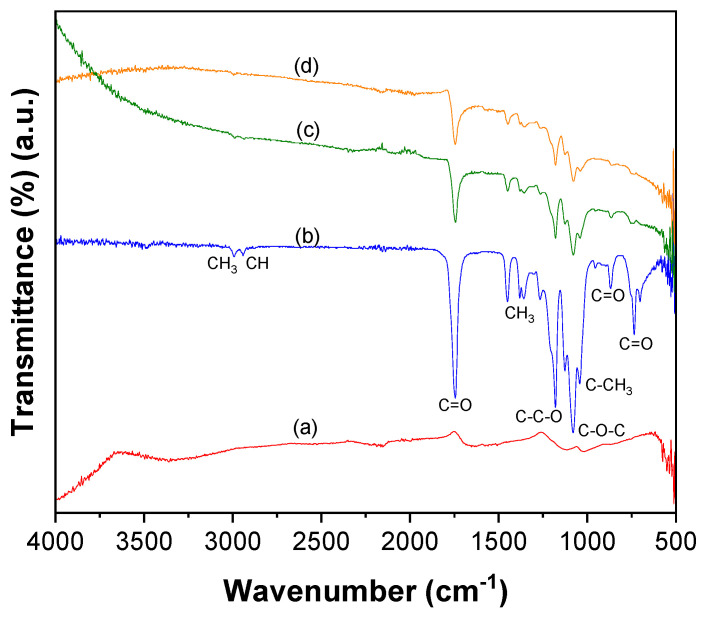
ATR–FTIR spectra of (**a**) CNTs–COOH, (**b**) PLA, (**c**) CNTs–PLA, and (**d**) CNTs–PLA–Pd.

**Figure 2:**
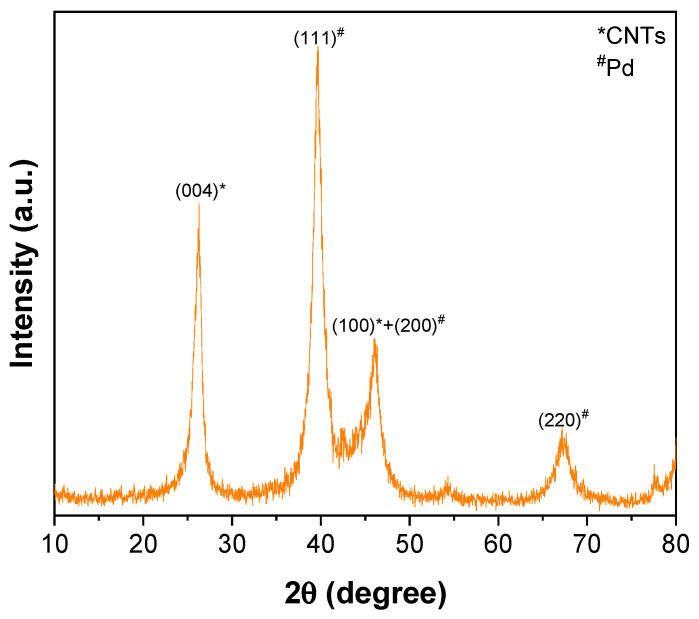
XRD of CNTs–PLA–Pd.

**Figure 3:**
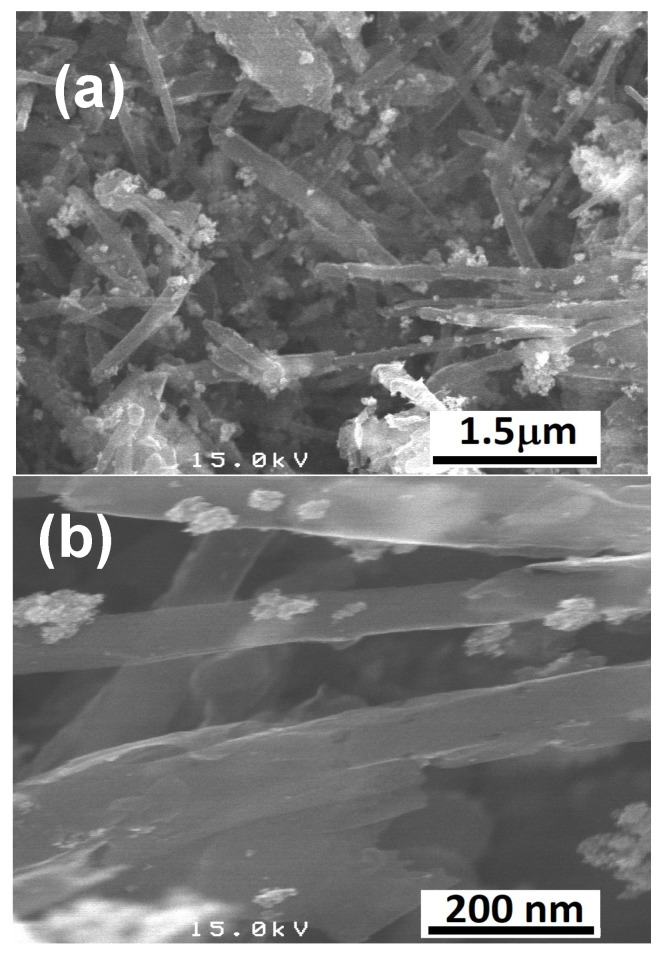
FESEM images of (**a**,**b**) CNTs–PLA–Pd.

The ATR–FTIR spectrum of CNTs–COOH (Figure 1a) exhibited the bands at 612 and 1634 cm^−1^ corresponding to the A2u and E1u phonon modes of CNTs [53]. It showed the band due to the O–H bonds at 3360 cm^−1^ and the band of C=O bonds of –COOH groups existing over the surface of CNTs at 1698 cm^−1^ [54]. The band of the C=C was found at 1559 cm^−1^ [54]. Compared to the spectra of CNTs–COOH and PLA (Figure 1b), CNTs–PLA (Figure 1c) displayed important characteristic bands. In particular, the bands that appeared at 734 and 864 cm^−1^ were due to C=O stretching, and the band at 1040 cm^−1^ was due to C–CH_3_ stretching [52,55]. The significant intensity band that appeared at 1080 cm^−1^ was due to C–O–C vibrations, and the absorption at 1126 cm^−1^ was owing to CH_3_ vibrations [55]. The band of C–C–O with high frequency was established at 1179 cm^−1^, and the band at 1262 cm^−1^ corresponds to C–H and C–O–C [52,55]. The doublet exists at 1355 and 1379 cm^−1^, and the singlet at 1449 cm^−1^ was assigned to CH_3_ vibrations. The band for C=O stretching was positioned at 1744 cm^−1^ with high intensity. The CH and CH_3_ vibrations band was displayed as a doublet at 2935 and 2988 cm^−1^, respectively. The absorption of –OH stretching was situated at 3500 cm^−1^. The spectrum of CNTs–PLA–Pd (Figure 1d) revealed the distinctive bands found in the spectrum of CNTs–PLA with diminished intensity and minute shifting in their position. The powder XRD of CNTs–PLA–Pd, presented in Figure 2, demonstrated the well–resolved reflection peaks at 26.4, 39.5, 45.8, 46.1, and 67.2°. The peaks located at 26.4 and 45.8° were assigned to (0 0 2) and (1 0 0) planes of CNTs, respectively [56]. The peaks situated at 39.5, 46.1, and 67.2° were due to the (1 1 1), (2 0 0), and (2 2 0) planes of Pd (JCPDS No. 89–4897). The FESEM images of CNTs–PLA–Pd, illustrated in Figure 3, show the effective exfoliation of CNTs. It was approached by the successful grafting of PLA chains to the surface of CNTs. The repulsion between the polymeric chains of PLA caused the efficient exfoliation of CNTs. The presence of individual CNT is observable in Figure 3a. However, it is perceptibly visible in Figure 3b. The CNTs have a width of about 50–75 nm and a length of several μm. Some CNTs have a distracting surface, which could be created by the harsh treatment of pristine CNTs with a mixture of concentrated sulfuric and nitric acids applied before the grafting of PLA. Furthermore, the existence of densely populated Pd nanoparticles over the surface of PLA–grafted CNTs is evident in Figure 3. Nonetheless, Pd nanoparticles have assembled and formed some clusters that led to the seeding of twinned or multiple–twinned nanoparticles, which commonly occur in palladium [52,57,58,59]. The grafting of PLA to CNTs has provided a suitable environment for depositing the Pd nanoparticles and directed their strong adherence. The resilient anchoring of Pd nanoparticles to the surface of PLA–grafted CNTs prevented them from detachment.

**Figure 4:**
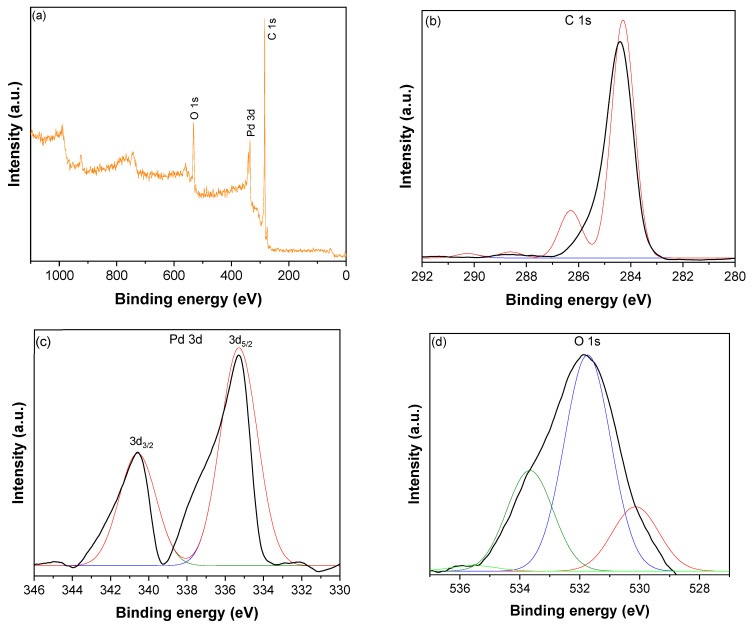
(**a**) XPS survey spectrum of CNTs–PLA–Pd, (**b**) The high–resolution spectrum of C1s, (**c**) The high–resolution spectrum of Pd 3d, and (**d**) The high–resolution spectrum of O 1 s.

The XPS survey spectrum of CNTs–PLA–Pd shown in Figure 4a revealed the presence of C, O, and Pd. The high–resolution spectrum of C1s (Figure 4b) displayed a peak at 284.3 eV, divulging into four distinct peaks by Gaussian fitting. The peak at 284.3 eV was assigned to C–C bonds of sp^2^ hybridized carbon atoms of CNTs [60,61]. The peak at 286.7 eV was due to C–C bonds occurring in structurally defective sp^3^ hybridized carbon atoms and C–O bonds of CNTs [60,61]. While the low–intensity peaks that appeared at 288.3 and 290.1 eV were owing to carbonyl, C=O, and carboxyl carbon O=C–O, respectively [60,61]. The spectrum of Pd 3d (Figure 4c) demonstrated the spin–orbit split doublet with a high–energy band of Pd 3d_3/2_ at 340.6 eV and a low–energy band of Pd 3d_5/2_ at 335.3 eV [62,63]. The difference in the binding energy between Pd 3d_3/2_ and Pd 3d_5/2_ peaks was 5.3 eV, which validates the occurrence of Pd in the zero–valent of the metallic state [60]. The deconvoluted O 1 s peaks (Figure 4d) show several types of oxygen species. The peak of lattice oxygen (O_2_^2−^) was situated at 530.1 eV. The peaks for C=O and C–O functional groups were located at 531.7 eV and 533.6 eV, respectively [64]. The peak at 535.5 eV is likely due to the chemisorbed oxygen or adsorbed molecular H_2_O [64].

**Figure 5:**
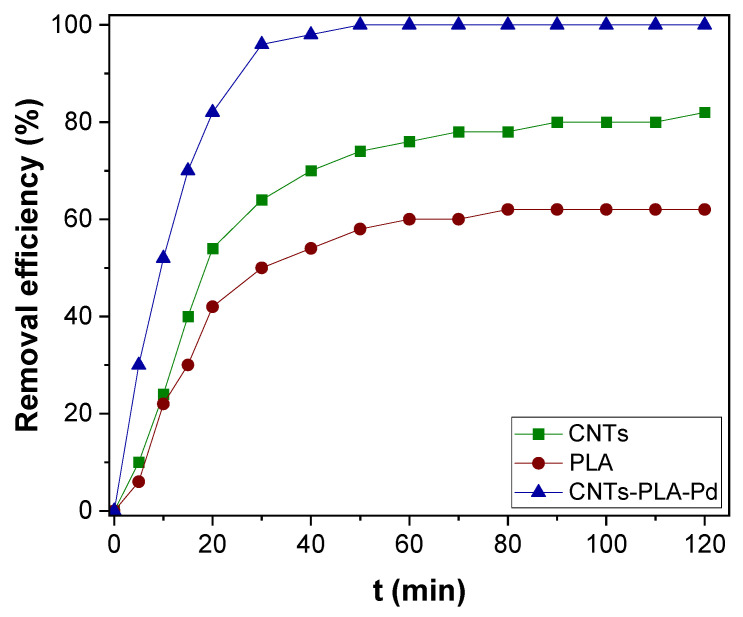
The adsorption kinetics of Hg(II) over CNTs, PLA, and CNTs–PLA–Pd.

**Figure 6:**
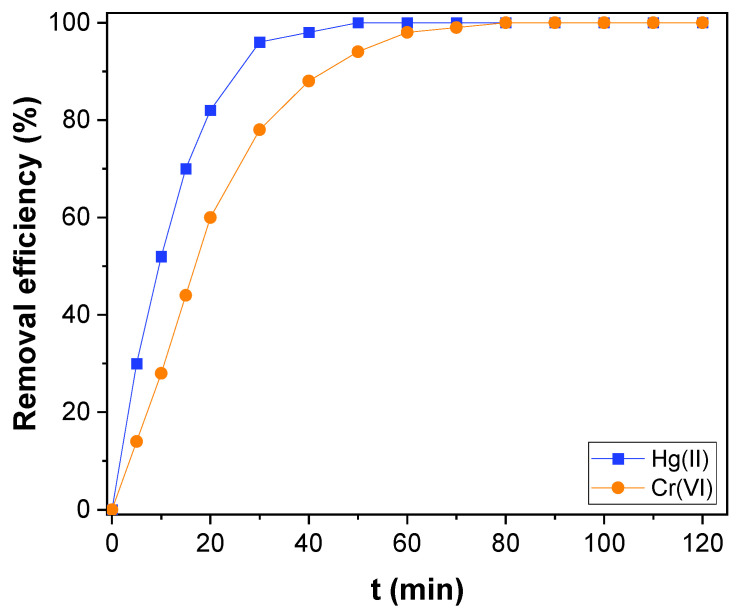
The adsorption kinetics of Hg(II) and Cr(VI) over CNTs–PLA–Pd.

The CNTs–PLA–Pd as adsorbent, its efficiency was revealed by evaluating the adsorption rate of Hg(II), and Cr(VI) present in water. It is known that the adsorption rate depends on the contact time between the adsorbent and adsorbate. So, the adsorption rate of Hg(II) was measured as a function of contact time and compared with CNTs and PLA (Figure 5). The CNTs–PLA–Pd was able to completely adsorb the Hg(II) within 50 min. However, CNTs and PLA were able to adsorb 74 and 58% of Hg(II), respectively. Therefore, the adsorption ability of CNTs and PLA was significantly improved after their blending and deposition of Pd nanoparticles in CNTs–PLA–Pd. Also, the efficiency of CNTs–PLA–Pd was estimated by the adsorption of Cr(VI) (Figure 6). The plot perceived for q_t_ versus t is shown in Figure S1. It represented that the adsorption rate of Hg(II) and Cr(VI) enhanced with contact time and finally red the equilibrium. Initially, the adsorption rate was high, followed by gradual reduction and final attainment of equilibrium. Within the initial 30 min, about 96 and 78% of Hg(II) and Cr(VI), respectively, were adsorbed. The complete adsorption of Hg(II) was accomplished in 50 min and Cr(VI) within 80 min. The rapid adsorption that occurred initially for 30 min could be due to the abundant availability of the active sites that exist over the surface of CNTs–PLA–Pd. With the progress of time, the active sites are being saturated by adsorption of the high quantity of Hg(II) and Cr(VI) ions and the repulsive forces transpire between solute molecules in the solid and bulk phases [65]. The results were analyzed by Pseudo–first and second–order models. The pseudo–first–order equation applied can be expressed as:

(4) $$n(q_{e}-q_{t})={\mathrm{lnq}}_{e}-k_{1}t$$

where q_e_ and q_t_ are the quantity of Hg(II) and Cr(VI) (mg g^−1^) at equilibrium and particular time t (min), respectively. k_1_ (min^−1^) is the pseudo–first–order rate constant. The slope of the plot, ln(q_e_ − q_t_) versus t, provides the value of k_1_, and the intercept gives the theoretical value of q_e_.

The pseudo–second–order equation used can be represented by:

(5) $$\frac{t}{q_{t}}=\frac{1}{k_{2}{\;q}_{e}^{2}}+\frac{t}{q_{e}}$$

where k_2_ [g (mg min)^−1^] is the second–order rate constant, which can be perceived by the plot of t/q_t_ against t. The slope is 1/q_e_, and the intercept is 1/k_2_ q_e_^2^.

**Figure 7:**
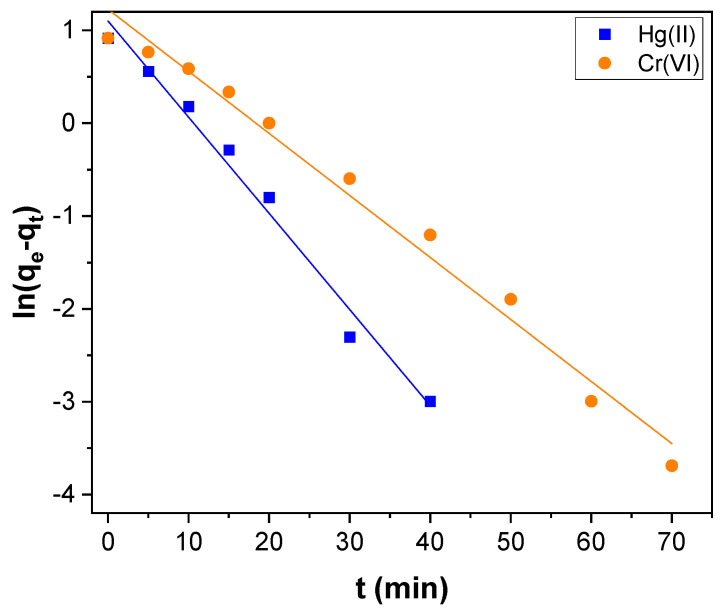
The pseudo–first–order kinetics for Hg(II) and Cr(VI) adsorption over CNTs–PLA–Pd.

**Figure 8:**
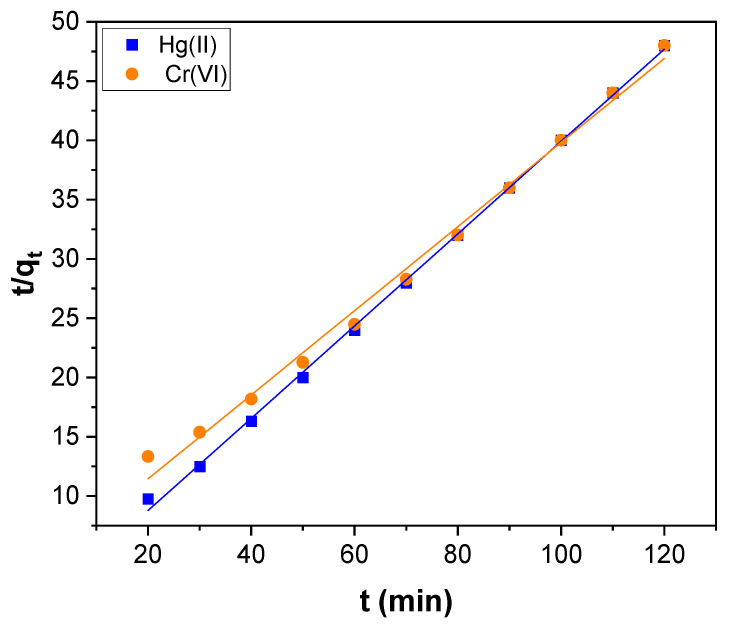
The pseudo–second–order kinetics for Hg(II) and Cr(VI) adsorption over CNTs–PLA–Pd.

The pseudo–first and second–order plots obtained for Hg(II) adsorption and Cr(VI) are shown in Figure 7 and Figure 8, respectively and the kinetic parameters determined are presented in Table 1. The correlation coefficient (R^2^) perceived for pseudo–first–order kinetics was 0.9850 and 0.9841 for Hg(II) and Cr(VI), respectively. For pseudo–second–order kinetics, it was 0.9991 and 0.9930 for Hg(II) and Cr(VI), respectively. The R^2^ value of pseudo–second–order kinetics was higher than first–order kinetics. Besides, the value of q_e_ (exp) agreed with the value of q_e_ (cal) estimated from pseudo–second–order kinetics rather than the value determined by pseudo–first–order kinetics for both Hg(II) and Cr(VI) (Table 1). Therefore, the Hg(II) and Cr(VI) adsorption could be better explained by pseudo–second–order kinetics rather than first–order kinetics. Hence chemisorption is the rate–limiting step in the Hg(II) and Cr(VI) adsorption over the surface of CNTs–PLA–Pd [66].

Further, reaction pathways and the rate–controlling step in the Hg(II) and Cr(VI) adsorption were evaluated by the application of the Weber−Morris intraparticle pore diffusion model using the following equation [67].

(6) $$q_{t}\;=k_{\mathrm{id}}{\;t}^{0.5}\;+\;c$$

where k_id_ (mg g^−1^min^−1^) is the intraparticle diffusion rate constant, which can be calculated from the linear plot of q_t_ versus t^0.5^ c (mg g^−1^) is the intraparticle diffusion constant, estimated from the intercept, and is directly proportional to the boundary layer thickness. It is assumed that the higher the intercept value, the more significant the contribution of the surface adsorption in the rate–controlling step. If the regression of q_t_ versus t^0.5^ plot is linear and passes through the origin, intraparticle diffusion plays a significant role in controlling the kinetics of the adsorption process. If it does not pass through the origin, in that case, intraparticle diffusion is not the only rate–limiting step. Instead, it is contributed by the boundary layer effect [68].

**Figure 9:**
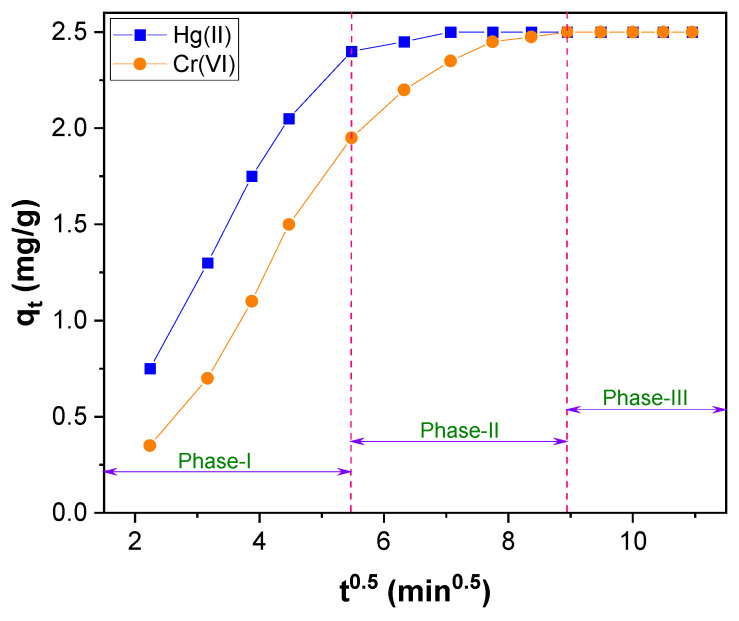
The Weber–Morris intraparticle diffusion plot for Hg(II) and Cr(VI) adsorption over CNTs–PLA–Pd.

The plot of q_t_ versus t^0.5^, shown in Figure 9, does not pass through the origin of the coordinates (Figure S2). So, intraparticle diffusion is not the sole rate–limiting step in the adsorption process of Hg(II) and Cr(VI). The intercept value obtained from Figure 9 was low (Table S1), and the intraparticle diffusion plot (Figure S2) revealed two straight lines. It shows that the intraparticle diffusion was not only a rate–controlling step in the Hg(II) and Cr(VI) adsorption; it was contributed by the boundary layer effect also [68,69]. The multilinearity of Figure 9 exhibits that the adsorption process of Hg(II) and Cr(VI) transpires through multiple phases instead of a single [66]. Among these multilinear steps, the initial phase occurred through the rapid adsorption of Hg(II) and Cr(VI) ions. The second phase could be the diffusion of Hg(II) and Cr(VI) ions into the pores of CNTs–PLA–Pd, and the third phase could be due to the equilibrium of adsorption that causes chemical reaction/bonding [66].

**Figure 10:**
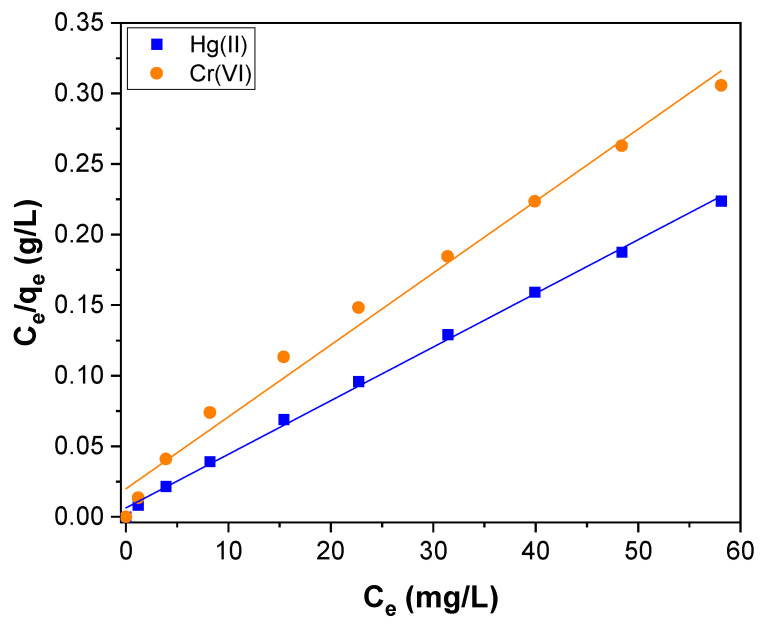
The Langmuir isotherm plot for Hg(II) and Cr(VI) adsorption over CNTs–PLA–Pd.

To reveal the Hg(II) and Cr(VI) adsorption in detail, the experimental equilibrium parameters were determined by three isotherm models viz., the Langmuir, the Freundlich, and the Temkin models. The Langmuir model assumes that the adsorbed molecules form a monolayer and that the adsorption can occur at a fixed number of adsorption sites and all equivalent in adsorption abilities. Each molecule also has a constant enthalpy and adsorption activation energy. In other words, all molecules have an equal affinity to entire adsorption sites [70]. With the help of the Langmuir isotherm model, it is possible to find the maximum adsorption capacity of the adsorbent using the linear form of the Langmuir isotherm model represented by the following equation:

(7) $$\frac{C_{e}}{q_{e}}=\frac{C_{e}}{q_{m}}+\frac{1}{K_{L\;}q_{m}}\;$$

where q_e_ (mg g^−1^) is the amount of adsorbed Hg(II) and Cr(VI) per unit mass of CNTs–PLA–Pd; C_e_ (mg L^−1^) is the concentration of Hg(II) and Cr(VI) at equilibrium; q_m_ is the maximum amount of the Hg(II) and Cr(VI) adsorbed per unit mass of CNTs–PLA–Pd to form a complete monolayer on the surface–bound at high C_e_. K_L_ is the Langmuir adsorption constant related to the free energy of adsorption. The linear fitting for the Langmuir plot of specific adsorption (C_e_/q_e_) versus the equilibrium concentration (C_e_) obtained for Hg(II) and Cr(VI) adsorption is shown in Figure 10. The parameters calculated by the Langmuir isotherm model are tabulated in Table 2. The maximum adsorption capacity (q_m_) calculated for the Hg(II) and Cr(VI) adsorption was 263.2 and 196.1 mg g^−1^, respectively. The value of K_L_ for Hg(II) and Cr(VI) was 0.6041 and 0.2567 L mg^−1^, respectively.

**Figure 11:**
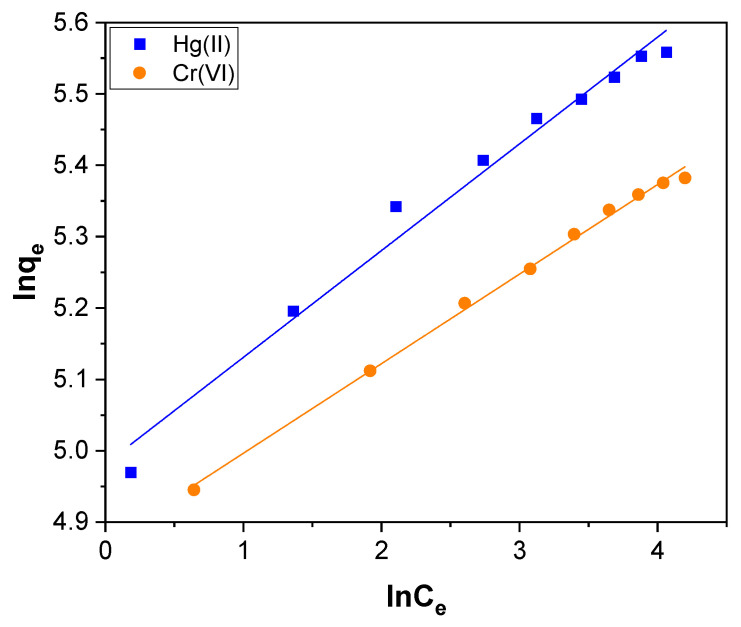
The Freundlich isotherm plot for Hg(II) and Cr(VI) adsorption over CNTs–PLA–Pd.

The Freundlich isotherm is derived from the assumption that the adsorption sites are distributed exponentially concerning the heat of adsorption [71]. It provides the relationship between the equilibrium of liquid and solid phase capacity based on the multilayer adsorption properties consisting of the heterogeneous surface of the adsorbent. The Freundlich isotherm, which supports multilayer adsorption, agrees with the Langmuir model over moderate ranges of concentrations but differs at low and high concentrations. The linear form of the Freundlich isotherm used is denoted by the below equation:

(8) $${\ln\;q}_{e}={\;\ln\;K}_{F}+\frac{{\ln\;C}_{e}}{n}$$

where q_e_ (mg g^−1^) is the amount of Hg(II) and Cr(VI) adsorbed at equilibrium. K_F_ and n are Freundlich constants. K_F_ symbolizes the affinity of the adsorbent, and n signifies the adsorption intensity. C_e_ (mg L^−1^) is the concentration of Hg(II) and Cr(VI) at equilibrium. The value of K_F_ and n were evaluated by the slope and intercept of the Freundlich isotherm plot shown in Figure 11, and the assessed values are presented in Table 2. The magnitude of the exponent, 1/n, indicates the favorability of adsorption. If the value of n ranging from 1 to 10 indicates the favorable conditions for the process of adsorption [72]. For the Hg(II) and Cr(VI) adsorption, the calculated value of n was 6.685 and 7.966, respectively. It reveals that Hg(II) and Cr(VI) adsorption over CNTs–PLA–Pd occurs in favorable conditions.

**Figure 12:**
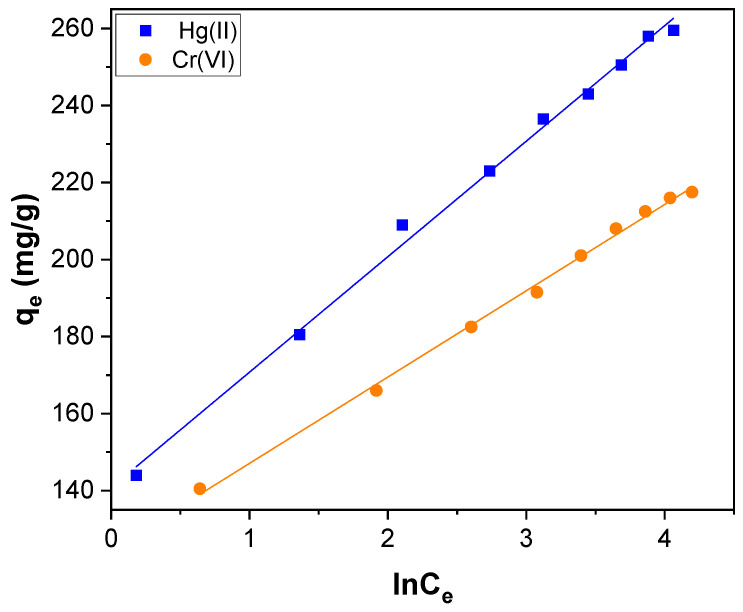
The Temkin isotherm plot for Hg(II) and Cr(VI) adsorption over CNTs–PLA–Pd.

The Temkin isotherm model assumes that during adsorption, the heat of all molecules decreases linearly with the increase in coverage of the adsorbent surface and that adsorption is characterized by a uniform distribution of binding energies up to maximum binding energy [73]. The Temkin isotherm model used is described by following equation:

(9) $${\;q}_{e}=B\;\mathrm{lnA}+{B\;\ln\;C}_{e}$$

where B = RT/K_T_, K_T_ is the Temkin constant related to the heat of adsorption (J mol^−1^); A is the Temkin isotherm constant (L g^−1^), R is the gas constant (8.314 J/mol K), and T the absolute temperature (K). The Temkin isotherm fitting plot of q_e_ versus ln C_e_ is shown in Figure 12, and the evaluated parameters are listed in Table 2.

All the tested Langmuir, Freundlich, and Temkin isotherm models have a better fit for Hg(II) and Cr(VI) adsorption with a high value of R^2^. In particular, for adsorption of Hg(II), R^2^_Lan_, R^2^_Fre,_ and R^2^_Tem_ were 0.9980, 0.9822, and 0.9959, respectively. Similarly, for adsorption of Cr(VI), R^2^_Lan_, R^2^_Fre,_ and R^2^_Tem_ were 0.9880, 0.9970, and 0.9970, respectively. Therefore, all three models are well–suited and validated with Hg(II) and Cr(VI) adsorption. It indicates that the adsorption mechanism of Hg(II) and Cr(VI) was associated with the basic assumption of all three isotherm models. In general, the Hg(II) and Cr(VI) adsorption over CNTs–PLA–Pd occurred through monolayer molecular covering and chemisorption together [74]. The maximum adsorption capacity (q_m_) of CNTs–PLA–Pd estimated for Hg(II) and Cr(VI) adsorption was compared to the reported values for different adsorbents and presented in Table 3 and Table 4, respectively [75,76,77,78,79,80,81,82,83]. The stability of CNTs–PLA–Pd was estimated by recovering and applying it in three subsequent cycles of adsorption of Hg(II). The adsorption capability of recycled CNTs–PLA–Pd was not significantly reduced in all four measured cycles. Therefore, CNTs–PLA–Pd is a sturdy adsorbent suitable for repeated use.

## 4. Conclusions

In conclusion, an efficient adsorbent, CNTs–PLA–Pd, was successfully produced by covalent grafting of PLA to CNTs and subsequent deposition of Pd nanoparticles. Thus, developed CNTs–PLA–Pd could remove Hg(II) and Cr(VI) entirely from the water within 50 min and 80 min, respectively. The initial rate for Hg(II) and Cr(VI) adsorption over CNTs–PLA–Pd was rapid, gradually reducing, and finally attained equilibrium. The analysis of experimental results revealed that the Hg(II) and Cr(VI) adsorption occurs through pseudo–second–order kinetics. It is identified that the chemisorption was the rate–limiting step in the adsorption process. The Weber−Morris intraparticle pore diffusion model exhibited that the adsorption of Hg(II) and Cr(VI) over CNTs–PLA–Pd occurs through multiple phases. The q_m_ value for Hg(II) and Cr(VI) adsorption was 263.2 and 196.1 mg g^−1^, respectively, and the K_L_ value was 0.6041 and 0.2567 L mg^−1^ for Hg(II) and Cr(VI) adsorption, respectively. The K_F_ value estimated for Hg(II) and Cr(VI) adsorption was 145.7 and 130.5 mg g^−1^, respectively, while the value of n was 6.685 and 7.966 for Hg(II) and Cr(VI) adsorption, respectively. The Langmuir, Freundlich, and Temkin models showed that the Hg(II) and Cr(VI) adsorption transpires with monolayer molecular covering and chemisorption. 

## Figures and Tables

**Table 1:** Parameters calculated for Hg(II) and Cr(VI) adsorption from adsorption kinetic models.

Adsorbate	q_e_ (exp) mg g^−1^	Pseudo–First Order Kinetic Model			Pseudo–Second–Order Kinetic Model		
		q_e_ (cal) mg g^−1^	k_1_ (min^−1^)	R^2^	q_e_ (cal) mg g^−1^	k_2_ (g mg^−1^ min^−1^)	R^2^
Hg(II)	2.500	1.0040	0.1036	0.9850	2.5686	0.1542	0.9991
Cr(VI)	2.500	1.2270	0.0668	0.9841	2.8190	0.0290	0.9930

**Table 2:** Parameters calculated for Hg(II) and Cr(VI) adsorption from Langmuir, Freundlich, and Temkin adsorption isotherms models.

Adsorbate	Langmuir Isotherm			Freundlich Isotherm			Temkin Isotherm		
	q_m_ (mg g^−1^)	K_L_ (L mg^−1^)	R^2^_Lan_	K_F_ (mg g^−1^)	n	R^2^_Fre_	A	B	R^2^_Tem_
Hg(II)	263.2	0.6041	0.9980	145.7	6.685	0.9822	109.1	30.00	0.9959
Cr(VI)	196.1	0.2567	0.9880	130.5	7.966	0.9970	260.5	22.41	0.9970

**Table 3:** Comparison of maximum adsorption capacity (q_m_) of CNTs–PLA–Pd estimated for Hg(II) adsorption with reported value for different adsorbents.

Adsorbent	Maximum Adsorption Capacity q_m_ (mg/g)	pH	Ref
RM–nZVI	94.58	5.0	[14]
UIO–66–NCS	250.0	5.0	[20]
UIO–66–IT	580.0	5.5	[20]
PAMC	76.3	6.0	[75]
CHAP–SH	282.7	4.5	[76]
M–PAL	203.4	4.0	[77]
ACM–5	191.9	5.8	[78]
MCS–4N	140.3	4.5	[79]
MCS–5N	109.7	4.5	[79]
CNTs–PLA–Pd	263.2	5.7	This work

**Table 4:** Comparison of maximum adsorption capacity (q_m_) of CNTs–PLA–Pd estimated for Cr(VI) adsorption with reported value for different adsorbents.

Adsorbent	Maximum Adsorption Capacity q_m_ (mg/g)	pH	Ref
BWAC	59.23	2.0	[7]
Chitosan/bentonite composite	16.38	3.0	[10]
NH_2_–ASNs	34.0	2.0	[24]
NH_2_–MSNs	42.2	2.0	[24]
Fe_3_O_4_ NPs	56.61	5.0	[80]
Oil palm bagasse	115.45	2.0	[81]
Yam peels	50.12	2.0	[81]
Al–GNSC	13.458	4.0	[82]
Cu (I)–MOF	96.0	6.0	[83]
CNTs–PLA–Pd	263.2	5.7	This work

## Data Availability

Not applicable.

## Supplementary Materials

The following supporting information can be downloaded at: https://www.mdpi.com/article/10.3390/toxics11060545/s1, Table S1: Parameters calculated from the intra-particle diffusion plot provided in Figure S2; Figure S1: The plot perceived q_t_ as a function of time for Hg(II) and Cr(VI) adsorption over CNTs–PLA–Pd; Figure S2: The Intraparticle diffusion model for Hg(II) and Cr(VI) adsorption over CNTs–PLA–Pd.

## References

[1] Khurshid H., Mustafa M.R.U., Isa M.H. (2022). Adsorption of chromium, copper, lead and mercury ions from aqueous solution using bio and nano adsorbents: A review of recent trends in the application of AC, BC, nZVI and MXene. Environ. Res..

[2] Foroutan R., Peighambardoust S.J., Ahmadi A., Akbari A., Farjadfard S., Ramavandi B. (2021). Adsorption mercury, cobalt, and nickel with a reclaimable and magnetic composite of hydroxyapatite/Fe_3_O_4_/polydopamine. J. Environ. Chem. Eng..

[3] Feng B., Shen W., Shi L., Qu S. (2018). Adsorption of hexavalent chromium by polyacrylonitrile–based porous carbon from aqueous solution. R. Soc. Open Sci..

[4] Raghunathan V.K., Tettey J.N.A., Ellis E.M., Grant M.H. (2009). Comparative chronic in vitro toxicity of hexavalent chromium to osteoblasts and monocytes. J. Biomed. Mater. Res. A.

[5] Cohen M.D., Kargacin B., Klein C.B., Costa M. (1993). Mechanisms of chromium carcinogenicity and toxicity. Crit. Rev. Toxicol..

[6] Alemu A., Lemma B., Gabbiye N. (2019). Adsorption of chromium (III) from aqueous solution using vesicular basalt rock. Cogent Environ. Sci..

[7] Dula T., Siraj K., Kitte S.A. (2014). Adsorption of hexavalent chromium from aqueous solution using chemically activated carbon prepared from locally available waste of bamboo (*Oxytenanthera abyssinica*). SRN Environ. Chem..

[8] Dzieniszewska A., Kyziol-Komosinska J., Pająk M. (2020). Adsorption and bonding strength of chromium species by ferrihydrite from acidic aqueous solutions. PeerJ.

[9] Kailasam S., Arumugam S., Balaji K., Kanth S.V. (2022). Adsorption of chromium by exopolysaccharides extracted from lignolytic phosphate solubilizing bacteria. Int. J. Biol. Macromol..

[10] Yang J., Huang B., Lin M. (2020). Adsorption of hexavalent chromium from aqueous solution by a chitosan/bentonite composite: Isotherm, kinetics, and thermodynamics studies. J. Chem. Eng. Data.

[11] Ayoub G.M., Damaj A., El-Rassy H., Al-Hindi M., Zayyat R.M. (2019). Equilibrium and kinetic studies on adsorption of chromium (VI) onto pine-needle-generated activated carbon. SN Appl. Sci..

[12] Zhu Y., Wu J., Wang H., Wang J., Shen H., Ying Z. (2021). Interference effect of experimental parameters on the mercury removal mechanism of biomass char under an oxy–fuel atmosphere. ACS Omega.

[13] Hu X., Yan L., Wang Y., Xu M. (2021). Ion–imprinted sponge produced by ice template–assisted freeze drying of salecan and graphene oxide nanosheets for highly selective adsorption of mercury (II) ion. Carbohydr. Polym..

[14] Sahu M.K., Patel R.K., Kurwadkar S. (2022). Mechanistic insight into the adsorption of mercury (II) on the surface of red mud supported nanoscale zero–valent iron composite. J. Contam. Hydrol..

[15] Zhang X., Hao Y., Wang X., Chen Z., Li C. (2016). Competitive adsorption of cadmium(II) and mercury(II) Ions from aqueous solutions by activated carbon from *xanthoceras sorbifolia* bunge hull. J. Chem..

[16] Chen X., Fang J., Liao S., Mia R., Li W., Gao C., Tian D., Li W. (2021). A smart chitosan nonwoven fabric coated with coumarin–based fluorophore for selective detection and efficient adsorption of mercury (II) in water. Sens. Actuators B.

[17] Tchounwou P.B., Yedjou C.G., Patlolla A.K., Sutton D.J. (2012). Heavy metal toxicity and the environment. Mol. Clin. Environ. Toxicol..

[18] Zhang Z., Wu D., Guo X., Qian X., Lu Z., Xu Q., Yang Y., Duan L., He Y., Feng Z. (2005). Visible study of mercuric ion and its conjugate in living cells of mammals and plants. Chem. Res. Toxicol..

[19] Park J.-D., Zheng W. (2012). Human exposure and health effects of inorganic and elemental mercury. J. Prev. Med. Public Health.

[20] Awad F.S., Bakry A.M., Ibrahim A.A., Lin A., El-Shall M.S. (2021). Thiol– and amine–incorporated UIO–66–NH_2_ as an efficient adsorbent for the removal of mercury(II) and phosphate ions from aqueous solutions. Ind. Eng. Chem. Res..

[21] Shen J., Zhang S., Zeng Z., Huang J., Shen Y., Guo Y. (2021). Synthesis of magnetic short–channel mesoporous silica SBA–15 modified with a polypyrrole/polyaniline copolymer for the removal of mercury Ions from aqueous solution. ACS Omega.

[22] Schroeder W.H., Munthe J. (1998). Atmospheric mercury—An overview. Atmos. Environ..

[23] Ma L., Han L., Chen S., Hu J., Chang L., Bao W., Wang J. (2019). Rapid synthesis of magnetic zeolite materials from fly ash and iron–containing wastes using supercritical water for elemental mercury removal from flue gas. Fuel Process. Technol..

[24] Jang E.-H., Pack S.P., Kim I., Chung S. (2020). A systematic study of hexavalent chromium adsorption and removal from aqueous environments using chemically functionalized amorphous and mesoporous silica nanoparticles. Sci. Rep..

[25] Kumar S., Nair R.R., Pillai P.B., Gupta S.N., Iyengar M.A.R., Sood A.K. (2014). Graphene oxide−MnFe_2_O_4_ Magnetic Nanohybrids for efficient removal of lead and arsenic from water. ACS Appl. Mater. Interfaces.

[26] Ramos-Hernández L.E., Pérez-Aguilar N.V., Ovando-Medina V.M., Oyervides-Muñoz E., Arcibar-Orozco J.A. (2022). Arcibar–Orozco, Photoinduced adsorption of Cr(VI) ions in nano–zinc oxide and nano–zinc oxide/polypyrrole composite. J. Appl. Polym. Sci..

[27] Liu H., Zhang Z., Shen F., Zhou Y., Liu J., Yang H. (2022). Two–dimensional WS_2_ as a new mercury removal material: Mercury conversion pathway and effect of defect. Fuel.

[28] Vicente-Martínez Y., Caravaca M., Soto-Meca A. (2021). Simultaneous adsorption of mercury species from aquatic environments using magnetic nanoparticles coated with nanomeric silver functionalized with L–Cysteine. Chemosphere.

[29] Bayuo J., Rwiza M.J., Sillanpa M., Mteia K.M. (2023). Removal of heavy metals from binary and multicomponent adsorption systems using various adsorbents—A systematic review. RSC Adv..

[30] Wan K., Wang G., Xue S., Xiao Y., Fan J., Li L., Miao Z. (2021). Preparation of humic acid/L-cysteine–codecorated magnetic Fe_3_O_4_ nanoparticles for selective and highly efficient adsorption of mercury. ACS Omega.

[31] Yang Y., Huang R., Xu W., Zhang J., Li C., Song J., Zhu T. (2021). Different crystal forms of ZnS nanomaterials for the adsorption of elemental mercury. Environ. Sci. Technol..

[32] Selvi K., Pattabhi S., Kadirvelu K. (2001). Removal of Cr(VI) from aqueous solution by adsorption onto activated carbon. Bioresour. Technol..

[33] Yang T., Han C., Tang J., Luo Y. (2020). Removal performance and mechanisms of Cr(VI) by an in-situ self-improvement of mesoporous biochar derived from chicken bone. Environ. Sci. Pollut. Res..

[34] Li L.-L., Feng X.-Q., Han R.-P., Zang S.-Q., Yang G. (2017). Cr(VI) removal via anion exchange on a silver–triazolate MOF. J. Hazard. Mater..

[35] Galán B., Castañeda D., Ortiz I. (2005). Removal and recovery of Cr(VI) from polluted ground waters: A comparative study of ion exchange technologies. Water Res..

[36] Kozlowski C.A., Walkowiak W. (2002). Removal of chromium(VI) from aqueous solutions by polymer inclusion membranes. Water Res..

[37] Adhoum N., Monser L., Bellakhal N., Belgaied J.-E. (2004). Treatment of electroplating wastewater containing Cu^2+^, Zn^2+^ and Cr(VI) by electrocoagulation. J. Hazard. Mater..

[38] Golbaz S., Jafari A.J., Rafiee M., Kalantary R.R. (2014). Separate and simultaneous removal of phenol, chromium, and cyanide from aqueous solution by coagulation/precipitation: Mechanisms and theory. Chem. Eng. J..

[39] Dupont L., Guillon E. (2003). Removal of hexavalent chromium with a lignocellulosic substrate extracted from wheat bran. Environ. Sci. Technol..

[40] Peng C., Meng H., Song S., Lu S., Lopez-Valdivieso A. (2004). Elimination of Cr(VI) from electroplating wastewater by electrodialysis following chemical precipitation. Sep. Sci. Technol..

[41] Kongsricharoern N., Polprasert C. (1996). Chromium removal by a bipolar electro–chemical precipitation process. Water Sci. Technol..

[42] Mulani K., Daniels S., Rajdeo K., Tambe S., Chavan N. (2013). Adsorption of chromium(VI) from aqueous solutions by coffee polyphenol–formaldehyde/acetaldehyde resins. J. Polym..

[43] Qu G., Zhou J., Liang S., Li Y., Ning P., Pan K., Ji W., Tang H. (2022). Thiol–functionalized multi–walled carbon nanotubes for effective removal of Pb(II) from aqueous solutions. Mater. Chem. Phys..

[44] Egbosiuba T.C., Egwunyenga M.C., Tijani J.O., Mustapha S., Abdulkareem A.S., Kovo A.S., Krikstolaityte V., Veksha A., Wagner M., Lisak G. (2022). Activated multi–walled carbon nanotubes decorated with zero valent nickel nanoparticles for arsenic, cadmium and lead adsorption from wastewater in a batch and continuous flow modes. J. Hazard. Mater..

[45] Wang Z., Xu W., Jie F., Zhao Z., Zhou K., Liu H. (2021). The selective adsorption performance and mechanism of multiwall magnetic carbon nanotubes for heavy metals in wastewater. Sci. Rep..

[46] Guldi D.M., Rahman G.M.A., Zerbetto F., Prato M. (2005). Carbon nanotubes in electron donor–acceptor nanocomposites. Accounts Chem. Res..

[47] Lin Y., Taylor S., Li H., Fernando K.A.S., Qu L., Wang W., Gu L., Zhou B., Sun Y.-P. (2004). Advances toward bioapplications of carbon nanotubes. J. Mater. Chem..

[48] Pathak A., Gupta B.D. (2021). Palladium nanoparticles embedded PPy shell coated CNTs towards a high performance hydrazine detection through optical fiber plasmonic sensor. Sens. Actuators B.

[49] Schroeder V., Savagatrup S., He M., Lin S., Swager T.M. (2019). Carbon nanotube chemical sensors. Chem. Rev..

[50] Silva M.M., Lopes P.E., Li Y., Pötschke P., Ferreira F.N., Paiva M.C. (2021). Polylactic acid/carbon nanoparticle composite filaments for sensing. Appl. Sci..

[51] Nagarajan V., Mohanty A.K., Misra M. (2016). Perspective on polylactic acid (PLA) based sustainable materials for durable applications: Focus on toughness and heat resistance. ACS Sustain. Chem. Eng..

[52] Neelgund G.M., Oki A. (2011). Pd nanoparticles deposited on poly(lactic acid) grafted carbon nanotubes: Synthesis, characterization, and application in Heck C–C coupling reaction. Appl. Catal. A Gen..

[53] Neelgund G.M., Aguilar S.F., Kurkuri M.D., Rodrigues D.F., Ray R.L. (2022). Elevated adsorption of lead and arsenic over silver nanoparticles deposited on poly(amidoamine) grafted carbon nanotubes. Nanomaterials.

[54] Neelgund G.M., Aguilar S.F., Jimenez E.A., Ray R.L. (2023). Adsorption efficiency and photocatalytic activity of silver sulfide nanoparticles deposited on carbon nanotubes. Catalysts.

[55] Yuniarto K., Purwanto Y.A., Purwanto S., Welt B.A., Purwadaria H.K., Sunarti T.C. (2016). Infrared and Raman studies on polylactide acid and polyethylene glycol–400 blend. AIP Conf. Proc..

[56] Neelgund G.M., Oki A. (2011). Photocatalytic activity of CdS and Ag_2_S quantum dots deposited on poly(amidoamine) functionalized carbon nanotubes. Appl. Catal. B Environ..

[57] Neelgund G.M., Oki A. (2020). Contribution of polylactic acid and Pd nanoparticles in the enhanced photothermal effect of carbon nanotubes. Chemistryselect.

[58] Xiong Y., Xia Y. (2007). Shape–controlled synthesis of metal nanostructures: The case of palladium. Adv. Mater..

[59] Winjobi O., Zhang Z., Liang C., Li W. (2010). Carbon nanzotube supported platinum–Palladium nanoparticles for formic acid oxidation. Electrochim. Acta.

[60] Wang Y., He Q., Ding K., Wei H., Guo J., Wang Q., O’Connor R., Huang X., Luo Z., Shen T.D. (2015). Multiwalled carbon nanotubes composited with palladium nanocatalysts for highly efficient ethanol oxidation. J. Electrochem. Soc..

[61] Li Y., Zhou W., Wang H., Xie L., Liang Y., Wei F., Idrobo J.-C., Pennycook S.J., Dai H. (2012). An oxygen reduction electrocatalyst based on carbon nanotube–graphene complexes. Nat. Nanotechnol..

[62] Mahendiran C., Rajesh D., Maiyalagan T., Prasanna K. (2017). Pd nanoparticles-supported carbon nanotube-encapsulated NiO/MgO composite as an enhanced electrocatalyst for ethanol electrooxidation in alkaline medium. Chemistryselect.

[63] She Y., Lu Z., Fan W., Jewell S., Leung M.K.H. (2014). Leung, Facile preparation of PdNi/rGO and its electrocatalytic performance towards formic acid oxidation. J. Mater. Chem. A.

[64] Tan H.T., Chen Y., Zhou C., Jia X., Zhu J., Chen J., Rui X., Yan Q., Yang Y. (2012). Palladium nanoparticles supported on manganese oxide–CNT composites for solvent-free aerobic oxidation of alcohols: Tuning the properties of Pd active sites using MnOx. Appl. Catal. B.

[65] Joshi S., Sharma M., Kumari A., Shrestha S., Shrestha B. (2019). Arsenic removal from water by adsorption onto iron oxide/nano-porous carbon magnetic composite. Appl. Sci..

[66] Zeng H., Zhai L., Qiao T., Yu Y., Zhang J., Li D. (2020). Efficient removal of As(V) from aqueous media by magnetic nanoparticles prepared with iron-containing water treatment residuals. Sci. Rep..

[67] Weber W.J., Morris J.C. (1963). Kinetics of adsorption on carbon from solution. J. Sanit. Eng. Div. Proceed. Am. Soc. Civ. Eng..

[68] Hameed B., Salman J., Ahmad A. (2009). Adsorption isotherm and kinetic modeling of 2,4-D pesticide on activated carbon derived from date stones. J. Hazard. Mater..

[69] Hamayun M., Mahmood T., Naeem A., Muska M., Din S., Waseem M. (2013). Equilibrium and kinetics studies of arsenate adsorption by FePO_4_. Chemosphere.

[70] Langmuir I. (1916). The constitution and fundamental properties of solids and liquids Part I. solids. J. Am. Chem. Soc..

[71] Freundlich H.M.F. (1906). Over the adsorption in solution. J. Phys. Chem..

[72] Ploychompoo S., Chen J., Luo H., Liang Q. (2020). Fast and efficient aqueous arsenic removal by functionalized MIL-100(Fe)/rGO/d-MnO_2_ ternary composites: Adsorption performance and mechanism. J. Environ. Sci..

[73] Temkin M.J., Pyzhev V. (1940). Recent modifications to Langmuir isotherms. Acta Physiochim. USSR.

[74] Tabuchi A., Ogata F., Uematsu Y., Toda M., Otani M., Saenjum C., Nakamura T., Kawasaki N. (2022). Granulation of nickel-aluminum–zirconium complex hydroxide using colloidal silica for adsorption of chromium(VI) ions from the liquid phase. Molecules.

[75] Al-Yaari M., Saleh T.A. (2022). Mercury Removal from water using a novel composite of polyacrylate-modified carbon. ACS Omega.

[76] Wang Q., Zhu S., Xi C., Jiang B., Zhang F. (2022). Adsorption and removal of mercury(II) by a crosslinked hyperbranched polymer modified via sulfhydryl. ACS Omega.

[77] Kang C., Gao L., Zhu H., Lang C., Jiang J., Wei J. (2021). Adsorption of Hg(II) in solution by mercaptofunctionalized palygorskite. Environ. Sci. Pollut. Res..

[78] Li Y., Li W., Liu Q., Meng H., Lu Y., Li C. (2018). Alkynyl carbon materials as novel and efficient sorbents for the adsorption of mercury(II) from wastewater. J. Environ. Sci..

[79] Shen W., Fang Y., Azeem M., Gao Y., Li X., Zhao P., Ali A., Li M., Li R. (2021). Chitosan crosslinked with polyamine-co-melamine for adsorption of Hg^2+^: Application in purification of polluted water. Int. J. Biol. Macromol..

[80] Geneti S.T., Mekonnen G.A., Murthy H.C.A., Mohammed E.T., Ravikumar C.R., Gonfa B.A., Sabir F.K. (2022). Biogenic synthesis of magnetite nanoparticles using leaf extract of thymus schimperi and their application for monocomponent removal of chromium and mercury ions from aqueous solution. J. Nanomater..

[81] Villabona-Ortíz A., Tejada-Tovar C., González-Delgado D. (2022). Elimination of chromium (VI) and nickel (II) ions in a packed column using oil palm bagasse and yam peels. Water.

[82] Vaddi D.R., Gurugubelli T.R., Koutavarapu R., Lee D.-Y., Shim J. (2022). Bio-stimulated adsorption of Cr(VI) from aqueous solution by groundnut shell activated carbon@Al embedded material. Catalysts.

[83] Qi H., Niu X., Wu H., Liu X., Chen Y. (2021). Adsorption of chromium (VI) by Cu (I)-MOF in water: Optimization, kinetics, and thermodynamics. J. Chem..

