# Supplementary material for: Facilitated Adsorption of Mercury(II) and Chromium(VI) Ions over Functionalized Carbon Nanotubes
Source: Toxics. 2023 Jun 20;11(6):545. doi: 10.3390/toxics11060545 (PMC10304972; doi:10.3390/toxics11060545)
Supplement: Supplementary file 1 [file toxics-11-00545-s001.zip › toxics-2308795-supplementary.pdf]

toxics

Supplementary Materials: Facilitated Adsorption of Mercury(II) and Chromium(VI) Ions over Functionalized Carbon Nanotubes

Gururaj M. Neelgund, Erica A. Jimenez, Ram L. Ray and Mahaveer D. Kurkuri

Table S1. Parameters calculated from the intra-particle diffusion plot provided in Figure S2. .

Adsorbent	Intra-particle diffusion model					
	K_{id-1} [mg/(g.min$^{0.5}$)]	c	R^2	k_{id-2} [mg/(g.min$^{0.5}$)]	c	R^2
Hg(II)	0.5177	-0.3402	0.9841	6.29x10^{-3}	2.4390	0.3532
Cr(VI)	0.4373	-0.5877	0.9793	1.39x10^{-2}	2.3586	0.6600

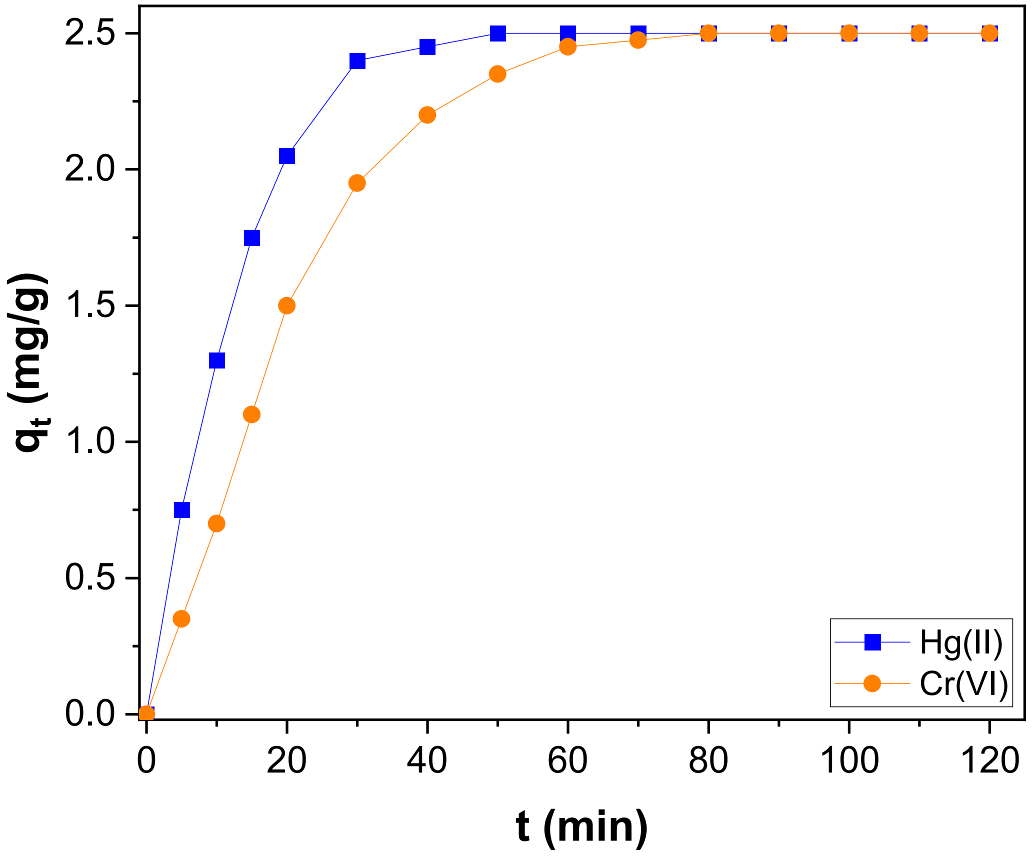

Figure S1. The plot perceived q_t as a function of time for Hg(II) and Cr(VI) adsorption over CNTs–PLA–Pd.

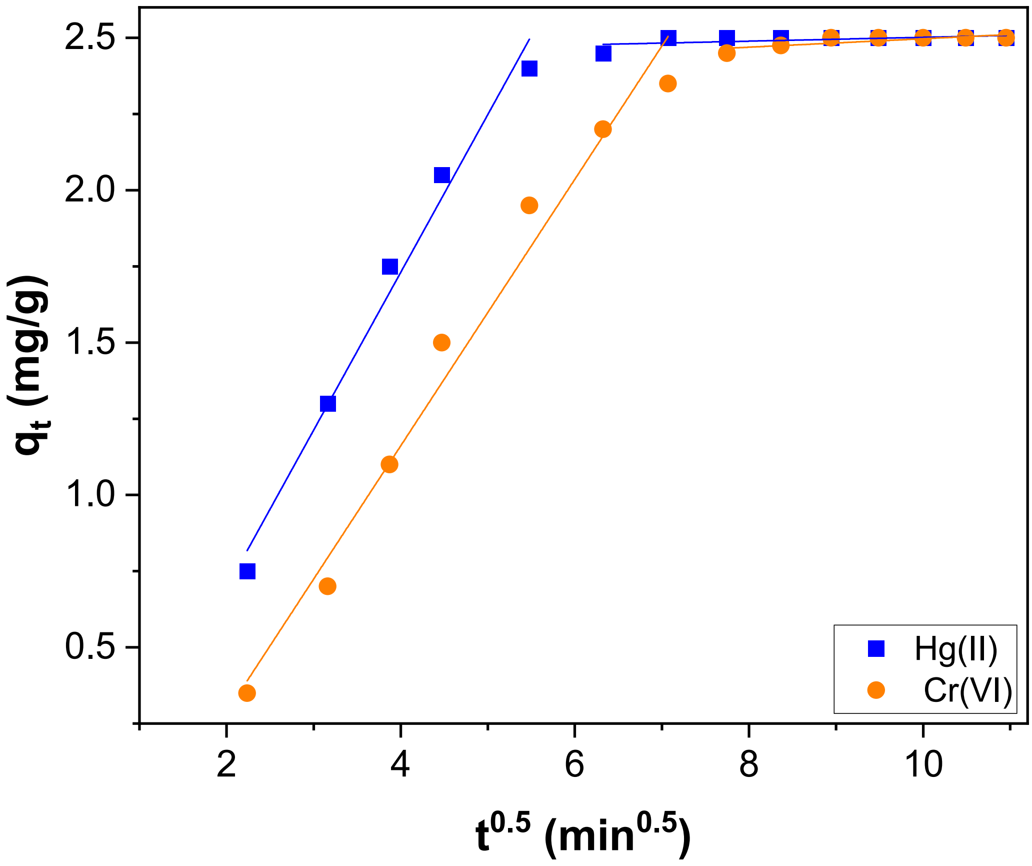

Figure S2. The Intraparticle diffusion model for Hg(II) and Cr(VI) adsorption over CNTs–PLA–Pd.
